# Morphofunctional Changes in Brain and Peripheral Blood in Adult and Aged Wistar Rats with AlCl_3_-Induced Neurodegeneration

**DOI:** 10.3390/biomedicines11092336

**Published:** 2023-08-22

**Authors:** Alexandra Vladislavovna Sentyabreva, Ekaterina Alexandrovna Miroshnichenko, Ekaterina Andreevna Melnikova, Ivan Sergeevich Tsvetkov, Anna Mikhailovna Kosyreva

**Affiliations:** 1 Avtsyn Research Institute of Human Morphology of “Petrovsky National Research Centre of Surgery”, 117418 Moscow, Russia; 2 Research Institute of Molecular and Cellular Medicine, Peoples’ Friendship University of Russia (RUDN University), 117198 Moscow, Russia

**Keywords:** aging, inflammation, inflammaging, neurodegeneration, animal models, Alzheimer’s disease

## Abstract

Background: the general lifespan has been prolonged greatly during the past century, and the incidence of age-associated diseases, including neurodegenerative ones, has increased as well. However, modelling of age-related pathologies is mostly conducted on adult rodents. We studied morphofunctional changes in the brain and peripheral blood of adult Wistar rats in comparison with old Wistar rats to determine age-related physiological changes and differences in adaptive reactions to AlCl_3_ exposure. Methods: the work was performed on adult and old male Wistar rats. The animals consumed a 100 mg/kg solution of AlCl_3_ each day for 60 days. Morphological changes of neurons and microglia, mRNA expression levels of pro-inflammatory and anti-inflammatory cytokines, microglia activation markers, amyloid-related proteins, and hallmarks of cellular senescence, monocyte, and lymphocyte subpopulations in the peripheral blood were examined. Results: old rats showed increasing hyperchromic neurons in the hippocampus; activation of microglia; upregulation of pro-inflammatory cytokines and cellular senescence markers; downregulation of anti-inflammatory cytokines; and *Hif-1a* and a decrease in B-cells and monocyte in peripheral blood. Conclusion: compared to young animals, aged rats respond to aluminum exposure with a severe decline of most cells’ function and irreversible neuronal loss. Regarding all reported data, neurodegeneration modelling and investigating of factors capable of accelerating or preventing it should be performed in experimental work on aged animals.

## 1. Introduction

Due to massive improvements in health care during the past century, the world’s population is growing—and aging. This leads to an increasing number of age-related diseases, including ones leading to dementia. Dementia is the most prevalent cause of disability globally [1] and is the outcome of various diseases, including neurodegenerative ones such as Alzheimer’s disease (AD). There were more than 55 million patients with dementia worldwide in 2019 [1] and, if this trend continues, there will be more than 150 million of them by 2050. This is a colossal burden not only for patients, their families, and healthcare and social workers. It is also a huge economic load on most countries, with an annual global cost of more than USD 1.3 trillion for treatment and social support [1]. Such great costs associated with neurodegenerative diseases and dementia at its terminal stage, especially caused by AD development, could be caused by an absence of any effective treatment so far. The FDA’s approved drugs can only faintly and briefly alleviate the symptoms.

There is still no consensus on the initial mechanisms of AD pathogenesis. Recent data showed that it is quite a complex process, not limited by amyloid deposits alone [2]. In the third part of a clinical trial of Lecanemab, an amyloid-antibody based drug, the drug showed rather low efficacy [3]. The impact of inflammaging, also known as systemic age-related chronic low-grade inflammation, is one of the hypotheses with the greatest potential to be investigated. It is a manifestation of senescence-associated secretory phenotype (SASP) which is expressed by senescent cells of aged organisms [4]. Inflammaging is one of the risk factors for the development of age-related diseases such as atherosclerosis, type 2 diabetes mellitus (T2DM), metabolic syndrome, etc. At the same time, these very pathological conditions, as well some others, like major depressive disorder [5], contribute to enhancing of its pro-inflammatory background.

The sporadic or late onset form of AD begins to manifest with mild cognitive impairment in people of age >60–65 years [6], which make it a part of the age-related pathologies group. The modeling of these pathologies, however, is still being conducted mostly on adult rodents, whereas experiments on old animals can provide more relevant data due to the presence of inflammaging. Furthermore, lots of studies are being performed on various lines of transgenic mice, which now seem not precisely relevant. The pathological processes of these mice do not exactly correspond with ones leading to neurodegeneration in humans. Within the wide variations of AD animal models, there are those based on exposure to aluminum compounds. Al^3+^ ions are capable of increasing the production of reactive oxygen species (ROS). ROS are involved in mitochondrial and DNA damage as well as in the promotion of the production of pro-inflammatory mediators and the establishment of a hypoxic condition [7,8]. All these events are typical for aging as well. Hence, the purpose of this work was to study morphofunctional changes of the brain and peripheral blood in adult Wistar rats in comparison with old Wistar rats to determine age-related physiological changes and differences in adaptive reactions to AlCl_3_ exposure.

## 2. Material and Methods

### 2.1. Animals

The work was performed on adult (n = 20, 3 months old) and old (n = 20, 24 months old) male Wistar rats. Animals were divided randomly into two experimental groups of adult (Adult-AlCl_3_) and old (Old-AlCl_3_) rats, and two control groups of adult (Adult-C) and old (Old-C) rats, comprising 10 animals each. Animals were kept in plastic cages (60 cm × 38 cm × 18.5 cm) in social groups of five; each animal was permitted free access to food and water. The temperature in the vivarium room was maintained within 18–22 °C, and air humidity was 50–65%. The study was approved by the Bioethical Commission of the Avtsyn Research Institute of Human Morphology of Petrovsky National Research Centre of Surgery (Protocol №36 (12) 28 March 2022). All experimental work involving animals was performed according to directive 2010/63/EU of the European Parliament and of the Council of the EU on the protection of animals used for scientific purposes (Strasbourg, 22 September 2010).

### 2.2. Neurodegeneration Modelling

Adult and old rats of both experimental groups consumed aluminum chloride (AlCl_3_) in a dosage of 100 mg/kg per day for 60 days with drinking water, as described previously [9]. Rats of both control groups consumed regular drinking water.

### 2.3. Cognitive Function Evaluation

Evaluation of cognitive function and, in particular, short-term memory consistency, was performed on day 0 (before consumption of AlCl_3_ began) and day 56 of the experiment using the Morris water maze (MWM) test as previously described [10]. Briefly, the Morris labyrinth was a grey-walled pool 150 cm in diameter and 60 cm of height, filled with water to a 40 cm height. The water temperature was 22 ± 2 °C. A round platform made of clear plastic (8 cm in diameter) was placed 1.5–2 cm below the water surface in the center of one of the pool quadrants. The pool was placed in a room with permanent spatial landmarks, and all test sessions were conducted under natural lightening. Animals were trained for one session per day for 2 continuous days. During the acquisition trials, rats were placed in the water from eight different points at approximately the same distance from the platform and each other on the one semicircle of the pool. After reaching the platform, the animal remained sitting on it for 15 s; then, the animal was placed in a separate cage for 60 s. Rats that did not find the platform within 60 s were gently guided towards it and left on it for 15 s as well. In each trial, the time required for every animal to reach the platform was recorded.

### 2.4. Samples Obtained and Histological Preparations

On the 61st day of the experiment, samples of peripheral blood were obtained under Zoletil (Vibrac Sante Animale, France) anesthesia; then, animals were euthanized by overdose (15 mg/kg) of Zoletil. The whole brains were fixed in 10% buffered formalin (BioVitrum, Saint-Petersburg, Russia) for 24 h, then dissected at the level of 6.0 mm posterior relative to bregma (each sample was 5 mm thick) [11]. After that, the specimens were dehydrated with ethanol of increasing concentration, cleared with xylene, infiltrated with a histological wax, and embedded in paraffin blocks for further slicing (5 µm thick).

### 2.5. Morphological Study

For morphological study, histological sections of brains were stained according to Nissl’s method. The absolute number of neurons in the standard area of the visual field (25,000 μm^2^) and the relative number of hyperchromic and morphologically altered neurons were evaluated on these sections in zones CA1, CA3, and in the dentate gyrus of the hippocampus. Images were captured using the Leica microscope (DM 2500 Leica Microsystems, Germany) on magnification ×400.

### 2.6. Immunohistochemical Study

For ICH-P study, frontal histological sections of brains (6.0 mm posterior relative to bregma) were prepared as previously described [12]. Then, they were stained with rabbit primary antibodies Iba1 (1:100; P4C288Ra01, Cloud Clone) + secondary HRP Donkey-anti-Rabbit antibody (1:500; 416,035, Novex Life Technologies) with additional hematoxylin staining. Images were captured using the Leica microscope (DM 2500 Leica Microsystems) on magnification ×1600.

### 2.7. qPCR-RT Study

The expression of mRNA was assayed by real-time qPCR in tissue fragments of the prefrontal cortex, preserved in IntactRNA solution (Eurogen, Russia), and stored at −20 °C until studied. The performed analysis included the detection and evaluation of expression levels of pro-inflammatory cytokines (*Il-6*, *Il-18*, and *Tnf-α*), anti-inflammatory cytokines (*Il-10* and *Tgf-β*), microglia M1 (*iNos*) and M2 (*Cd163*) activation markers, amyloid-related proteins (*App* and *Bace1*), and markers of cellular senescence (*Mmp9*, *Hif-1a*, *p16*, and *SA-β-galactosidase*). The measured mRNA expression levels, relative to the expression level of the gene *Gapdh* as a reference [13], were determined using qPCRmix-HS SYBR (Eurogen, Russia) with fluorescent intercalating dye SYBR Green I. Amplification, detection, and digital analysis of fluorescence levels in real time was performed on DT-96 Real-Time PCR Cycler (DNA-Technology JSC, Moscow, Russia) in standard mode at 95 °C for 5 min followed by 95 °C for 15 s, 62 °C for 10 s + reading, and 72 °C for 20 s ×45. All the primers’ sequences were picked up precisely for rat species by on-line soft Primer-BLAST (Appendix A).

### 2.8. Flow Cytometry

The relative numbers of lymphocyte subpopulations and monocyte in peripheral blood were counted using flow cytometry (Beckman Coulter, CA, USA). The following antibodies (eBioscience, San Diego, CA, USA; all anti-rat) were used for immune phenotypic analysis: CD3^+^-PE for examination of general T-lymphocyte population percentage; CD3^+^-PE + CD4^+^-FITC for CD3^+^CD4^+^ T-helpers; CD3^+^-PE + CD8-PE-Cy5 for CD3^+^CD8^+^ for T-cytotoxic cells; CD45R-FITC for CD45R^+^ B-cells; CD43-PE for CD43^+^ monocyte. Erythrocytes were lysed using the OptiLyse C solution (eBioscience, San Diego, CA, USA).

### 2.9. Statistical Analysis

The results were analyzed by Statistica 8.0 software (StatSoft, Inc., Tulsa, OK, USA). The normality of data distribution was checked by using the Kolmogorov–Smirnov test. The Kruskal–Wallis test and, post hoc, the Dunn test were used to establish the reliability of differences between groups by median. *p* < 0.05 was considered as statistically significant.

## 3. Results

### 3.1. Short-Term Memory Evaluation

**Figure 1:**
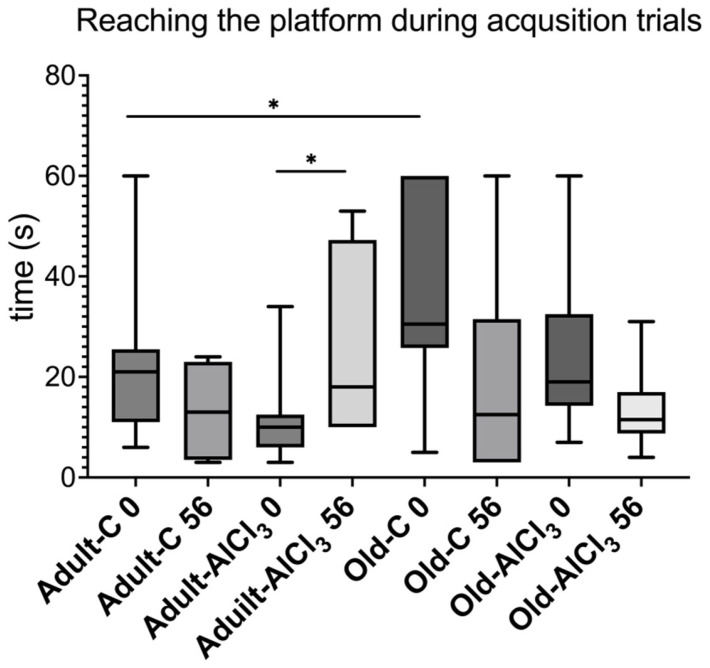
Time (s) required to reach the platform during acquisition trials for adult and old Wistar rats of control and experimental groups on day 0 and day 56 of the experiment. Adult-C 0—adult rats control group (n = 8), the trial on day 0 of the experiment; Adult-C 56—adult rats control group (n = 8), the trial on day 56 of the experiment; Adult-AlCl_3_ 0—adult rats that consumed AlCl_3_ (n = 8), the trial on day 0 of the experiment; Adult-AlCl_3_ 56—adult rats that consumed AlCl_3_ (n = 8), the trial on day 56 of the experiment; Old-C 0—old rats control group (n = 8), the trial on day 0 of the experiment; Old-C 56—old rats control group (n = 8), the trial on day 56 of the experiment; Old-AlCl_3_ 0—old rats that consumed AlCl_3_ (n = 8), the trial on day 0 of the experiment; Old-AlCl_3_ 56—old rats that consumed AlCl_3_ (n = 8), the trial on day 56 of the experiment. The data displayed as: line—median, box—25–75 quartiles, whiskers—nonoutlier range; *—*p* < 0.05. The Kruskal–Wallis test was used for multiple comparisons.

During the acquisition trials performed before the beginning of the experiment (day 0), Old-C rats took 1.5-fold more time to reach the platform in MWM than Adult-C ones. But there was no difference between these groups when the test was carried out on the 56th day of the experiment. Adult-AlCl_3_ rats required 1.8-fold more time to perform the task after 2 months of AlCl_3_ treatment, in comparison to the time required before treatment began (Figure 1).

### 3.2. The Percentage of Hyperchromic Neurons

**Figure 2:**
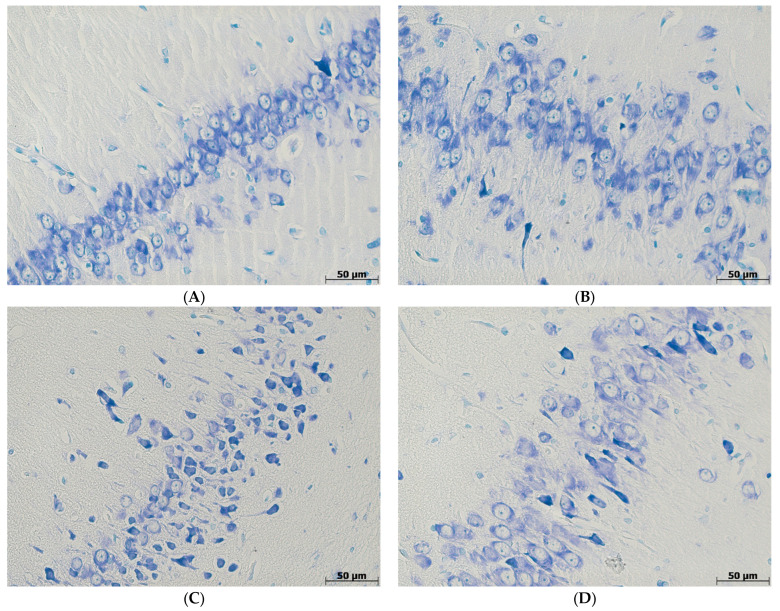

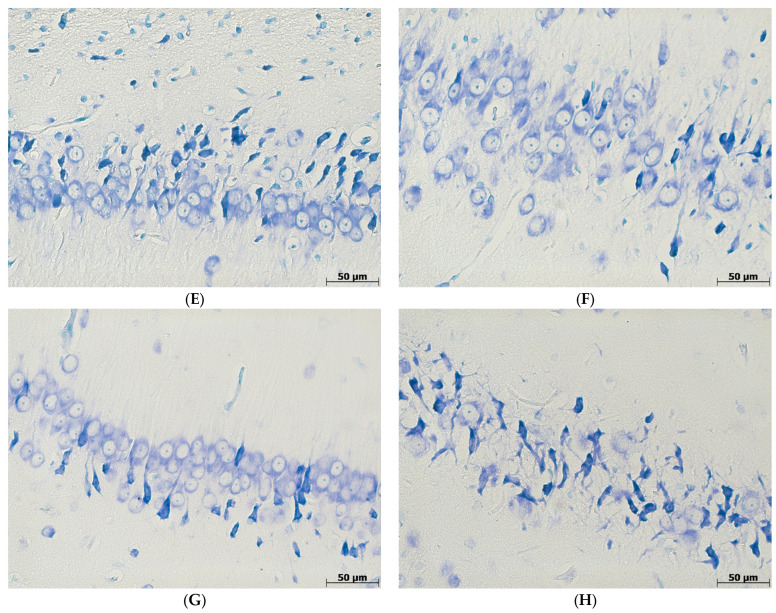
CA1 (**A**,**C**,**E**,**G**) and CA3 (**B**,**D**,**F**,**H**) zones of the hippocampus in rats of Adult-C group (**A**,**B**), Adult-AlCl_3_ group (**C**,**D**), Old-C group (**E**,**F**), and Old-AlCl_3_ group (**G**,**H**). Nissl’s staining, ×400.

**Figure 3:**
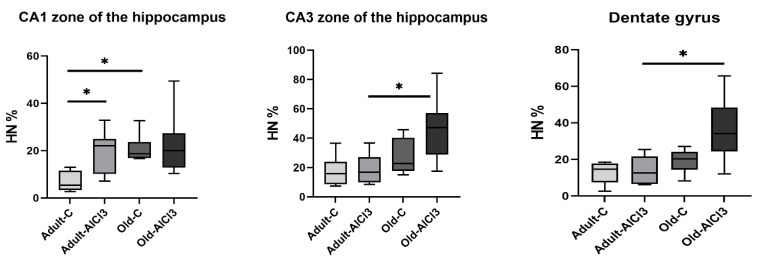
The percentage of hyperchromic neurons in zones CA1, CA3, and in the dentate gyrus of the hippocampus in adult and old Wistar rats of control and experimental groups. HN—hyperchromic neurons; Adult-C—adult rats control group (n = 8); Adult-AlCl_3_—adult rats that consumed AlCl_3_ (n = 8); Old-C—old rats control group (n = 8); Old-AlCl_3_—old rats that consumed AlCl_3_ (n = 8). The data displayed as: line—median, box—25–75 quartiles, whiskers—nonoutlier range; *—*p* < 0.05. The Kruskal–Wallis test was used for multiple comparisons.

The absolute numbers of neurons in zones CA1, CA3, and in the dentate gyrus of the hippocampus were nearly the same in all four groups of animals, whereas the relative numbers of altered hyperchromic neurons differed significantly among them. Old-C animals demonstrated a 3.5 times higher percentage of neurons in the CA1 hippocampal zone compared with Adult-C rats. This tended to increase in the CA3 zone and the dentate gyrus, but the difference between these data was statistically insignificant. Comparing adult animals, the hyperchromic neurons’ percentage was 4.1-fold higher in Adult-AlCl_3_ rats than in Adult-C rats, but only, as well, in the CA1 zone of the hippocampus. Meanwhile, Old-AlCl_3_ rats showed a 2.2-fold higher number of hyperchromic neurons in the CA3 hippocampal zone and a 2.8-fold higher number in the dentate gyrus compared with the Adult-AlCl_3_ group (Figure 2 and Figure 3).

### 3.3. Morphological Features of Microglia

**Figure 4:**
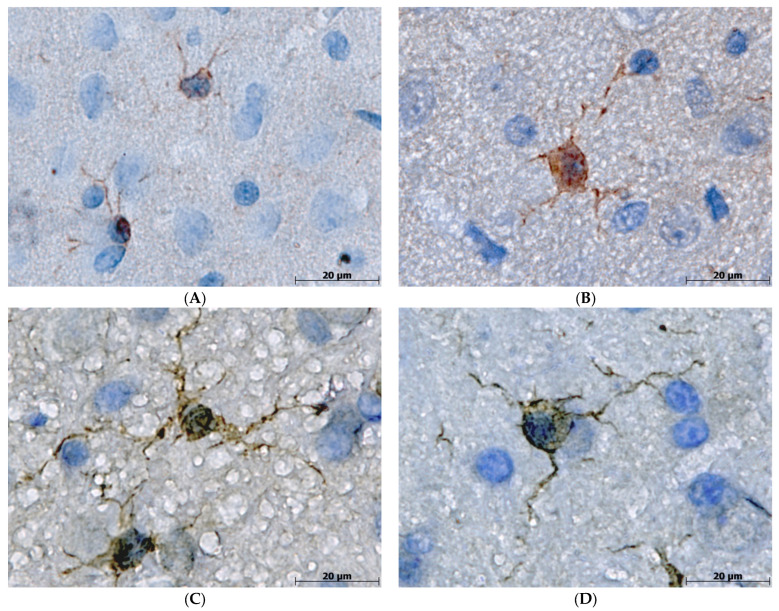
Morphological characteristics of microglia cells with thin and short processes in both Adult-C (**A**) and Adult-AlCl_3_ (**B**) groups; enlarged microglia with spheroidal swelling, hypertrophic, beaded, and tortuous processes in Old-C rats (**C**); and microglia of an increased size with beaded, tortuous, and fragmented but not thickened processes in Old-AlCl_3_ rats (**D**). Iba-1 antibody + HRP secondary antibody IHC and hematoxylin staining, ×1600. Adult-C—adult rats control group (n = 8); Adult-AlCl_3_—adult rats that consumed AlCl_3_ (n = 8); Old-C—old rats control group (n = 8); Old-AlCl_3_—old rats that consumed AlCl_3_ (n = 8).

Identified by ICH staining with anti-Iba1 antibody, the vast majority of microglial cells in the Adult-C group had a regular size of about 15–30 µm and thin, moderately ramified processes, which are features of the resting functional state. Compared with Adult-C rats’ microglial cells, those in the Old-C group had an increased size (>30 µm) and spheroidal, swollen, hypertrophic, beaded, and tortuous processes. In the Adult-AlCl_3_ group, microglia had the same morphological features as in the corresponding Adult-C group. At the same time, there were microglia of an increased size (>30 µm) concurrent with beaded, tortuous, and fragmented but not thickened processes in Old-AlCl_3_ rats, which distinguished them from both Adult-AlCl_3_ and Old-C group rats (Figure 4).

### 3.4. qPCR-RT Examination of the Prefrontal Cortex

#### 3.4.1. Pro-inflammatory Markers

The result of qPCR-RT of the prefrontal cortex tissue fragments showed that the pro-inflammatory cytokine *Il-18* expression level was higher in Old-C rats than in Adult-C ones by 74 fold. Notably, it was quite low and almost the same in both Adult-C and Adult-AlCl_3_ groups. But it also was higher, by 22 times, in Old-AlCl_3_ rats compared to Adult-AlCl_3_ rats. Compared with the Old-C group, there was a tendency for *Il-18* expression to decrease in the Old-AlCl_3_ group, although a statistically significant difference was not observed.

**Figure 5:**
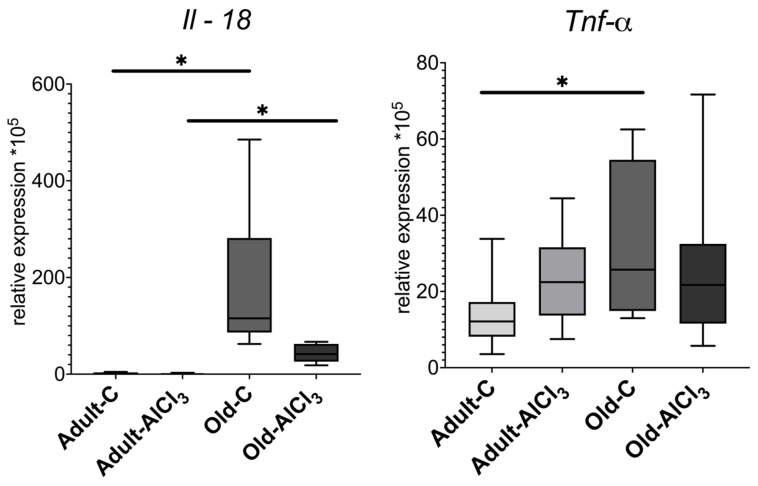
Mrna expression levels of *Il-18* and *Tnf-α* in the prefrontal cortex of adult and old Wistar rats in control and experimental groups. Adult-C—adult rats control group (n = 7); Adult-AlCl_3_—adult rats that consumed AlCl_3_ (n = 8); Old-C—old rats control group (n = 8) Old-AlCl_3_—old rats that consumed AlCl_3_ (n = 8). The data displayed as: line—median, box—25–75 quartiles, whiskers—nonoutlier range; *—*p* < 0.05. The Kruskal–Wallis test was used for multiple comparisons.

The level of *Tnf-α* expression was 2-fold higher in Old-C animals in comparison with Adult-C rats. Due to aluminum exposure, it increased 1.8-fold in Adult-AlCl_3_ rats compared with Adult-C ones, whereas it was measured at the same level in Old-AlCl_3_ rats as in both Adult-AlCl_3_ and Old-C groups (Figure 5).

#### 3.4.2. Anti-Inflammatory Markers

Unlike Adult-C rats, Old-C ones did not demonstrate a detectable expression level of anti-inflammatory cytokine *Il-10* at all. The Adult-AlCl_3_ group showed a decrease in its expression level by 3.1 times in comparison with the Adult-C group, whilst no *Il-10* expression level was observed in the Old-AlCl_3_ group or in the Old-C group.

**Figure 6:**
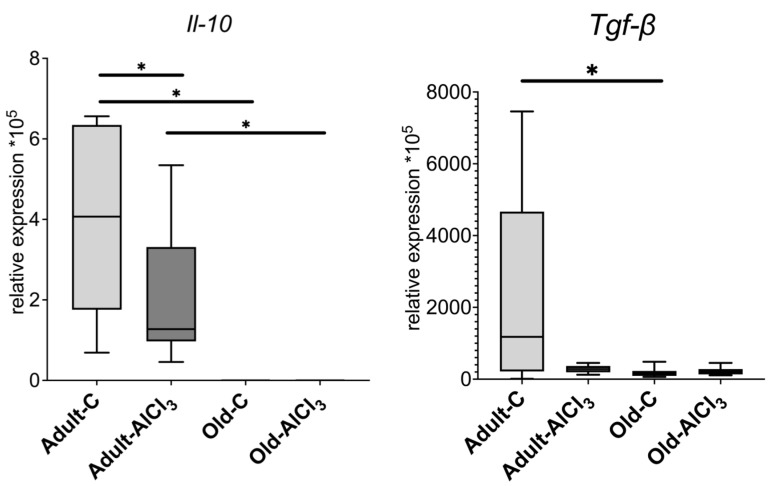
mRNA expression levels of *Il-10* and *Tgf-β* in the prefrontal cortex of adult and old Wistar rats in control and experimental groups. Adult-C—adult rats control group (n = 7); Adult-AlCl_3_—adult rats that consumed AlCl_3_ (n = 8); Old-C—old rats control group (n = 8); Old-AlCl_3_—old rats that consumed AlCl_3_ (n = 8). The data displayed as: line—median, box—25–75 quartiles, whiskers—nonoutlier range; *—*p* < 0.05. The Kruskal–Wallis test was used for multiple comparisons.

The expression level of *Tgf-β*, which is another marker of anti-inflammatory response, was 7.25 times less in the Old-C group compared with the Adult-C one. Also, compared with Adult-C rats, its expression level was downregulated notably in Adult-AlCl_3_ animals, although a statistically significant difference was not confirmed. The Old-AlCl_3_ group demonstrated a similar tendency of changing *Tgf-β* expression; as in the case of *Il-10* expression, its level was the same as that in the Old-C group (Figure 6).

#### 3.4.3. Microglia Activation Markers

The expression level of *iNos*, which is a pro-inflammatory cytokine and one of the markers of microglia activated towards M1 state, was 5.3-fold higher in Old-C rats in comparison with Adult-C ones. No difference of it was observed in the Adult-AlCl_3_ group compared with the Adult-C group. Likewise, the statistical difference of the *iNos* expression level in Old-AlCl_3_ animals relative to both Adult-AlCl_3_ and Old-C groups was unreliable.

Being a marker of M2 activated microglia, *Cd163* demonstrated a similar tendency as *iNos*, regarding changes in its expression level, and was upregulated by 8.5 times in Old-C animals relative to Adult-C ones. In Adult-AlCl_3_ rats, its expression level also slightly increased in comparison with Adult-C ones, with statistical difference unconfirmed. Notably, unlike the *iNos* expression level, the *CD163* level demonstrated down-regulation in Old-AlCl_3_ rats relative to both Adult-AlCl_3_ and Old-C groups; similarly, a statistical difference was unconfirmed.

**Figure 7:**
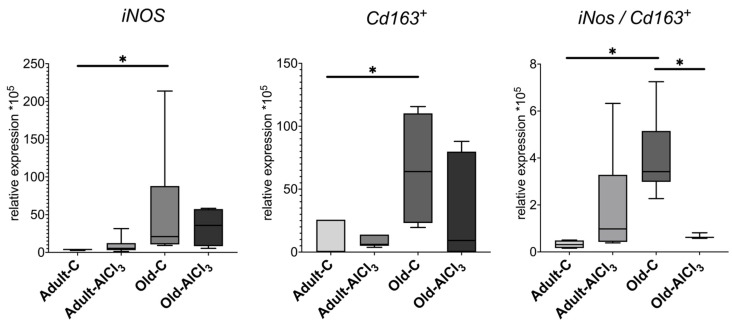
mRNA expression levels of *iNos* and *Cd163^+^* and M1/M2 (*iNos*/*Cd163^+^*) ratio in the prefrontal cortex in adult and old Wistar rats of control and experimental groups. Adult-C—adult rats control group (n = 7); Adult-AlCl_3_—adult rats that consumed AlCl_3_ (n = 8); Old-C—old rats control group (n = 6); Old-AlCl_3_—old rats that consumed AlCl_3_ (n = 5); The data displayed as: line—median, box—25–75 quartiles, whiskers—nonoutlier range; *—*p* < 0.05. The Kruskal–Wallis test was used for multiple comparisons.

Notably, the M1/M2 ratio calculated from the *iNos/Cd163* data of corresponding animals was 11-fold higher in the Old-C group in comparison with that of Adult-C rodents. Also, compared with Adult-C animals, this index was similar to the Adult-AlCl_3_ group. For Old-AlCl_3_ rats, this ratio declined by 5.5 times relative to Old-C rats but remained at the same level as that in the Adult-AlCl_3_ group (Figure 7).

#### 3.4.4. Amyloid-Related Proteins

Amyloid precursor protein (*App*) expression remained almost the same in Old-C rats compared with Adult-C ones, and no difference was observed between Adult-C and Adult-AlCl_3_ groups. However, the *App* expression level was 1.9 times less in the Old-AlCl_3_ group than in the Old-C one; this difference was not observed in the Adult-AlCl_3_ group.

**Figure 8:**
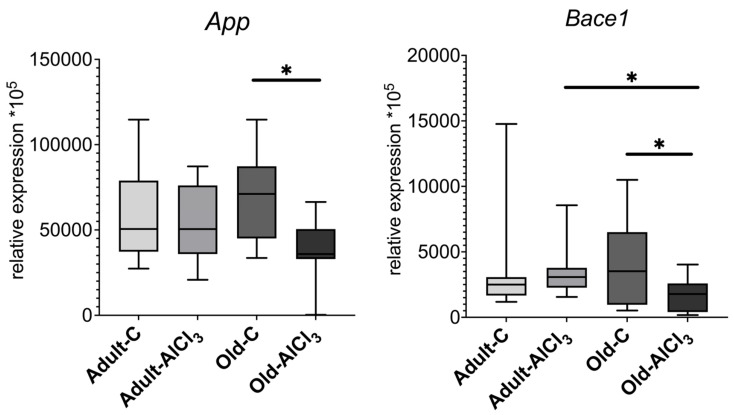
mRNA expression levels in *App* and *Bace1* in the prefrontal cortex of adult and old Wistar rats of control and experimental groups. Adult-C—adult rats control group (n = 7); Adult-AlCl_3_—adult rats that consumed AlCl_3_ (n = 8); Old-C—old rats control group (n = 8); Old-AlCl_3_—old rats that consumed AlCl_3_ (n = 8); The data displayed as: line—median, box—25–75 quartiles, whiskers—nonoutlier range; *—*p* < 0.05. The Kruskal–Wallis test was used for multiple comparisons.

Like *App*, Beta-site APP-cleaving enzyme 1 (*Bace1*) expression changed in a similar way when comparing Adult-C rats and Old-C rats as well as Adult-C and Adult-AlCl_3_ animals. However, it demonstrated significant downregulation in the Old-AlCl_3_ group relative to both the Old-C (2 times less) and the Adult-AlCl_3_ (1.7 times less) ones (Figure 8).

#### 3.4.5. Cellular Senescence Markers

Cyclin-dependent kinase inhibitor 2A, or p16 INK4a (*p16*), is a SASP marker, and its level of expression was upregulated by 10 times in Old-C rats in comparison with Adult-C ones. Relative to the Adult-C group, its level of expression remained the same in the Adult-AlCl_3_ group. The level of *p16* expression was the same in the Old-AlCl_3_ group as in the Old-C group.

The senescence-associated beta-galactosidase (*Sa-β-gal*) level of expression increased by 1.5 times in Old-C rats compared with Adult-C ones, whereas it did not change in the Adult-AlCl_3_ group relative to the Adult-C one. Old-AlCl_3_ rats showed the same *Sa-β-gal* expression level as both Old-C and Adult-AlCl_3_ animals.

The expression level of matrix metalloprotease 9 (*Mmp9*), which is a marker of both M2 microglia activation and SASP, was 3-fold more in the Old-C group than in Adult-C rodents. In Adult-AlCl_3_ rats, it remained the same as in Adult-C ones.

**Figure 9:**
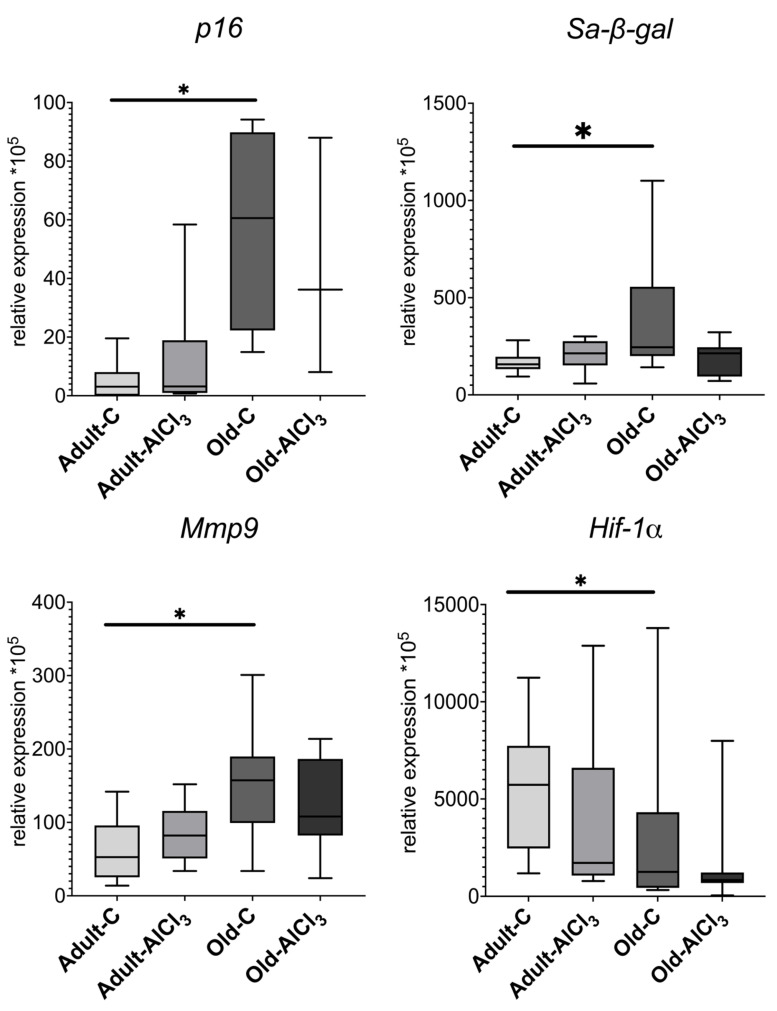
mRNA expression levels of *p16*, *Sa-β-gal*, *Mmp9*, and *Hif-1α* in the prefrontal cortex in adult and old Wistar rats of control and experimental groups. Adult-C—adult rats control group (n = 5); Adult-AlCl_3_—adult rats that consumed AlCl_3_ (n = 6); Old-C—old rats control group (n = 8); Old-AlCl_3_—old rats that consumed AlCl_3_ (n = 5); The data displayed as: line—median, box—25–75 quartiles, whiskers—nonoutlier range; *—*p* < 0.05. The Kruskal–Wallis test was used for multiple comparisons.

Hypoxia-inducible factor 1-alpha (*Hif-1a*) demonstrates the presence of a hypoxic condition and always appears alongside inflammation. Its expression level was 2.7-fold higher in the Adult-C group than in the Old-C one. In Adult-AlCl_3_ rats, it demonstrated a steady tendency of downregulation in comparison with Adult-C ones, with statistical difference unconfirmed. Old-AlCl_3_ rats showed the same level of it as those in both Adult-AlCl_3_ and Old-C groups (Figure 9).

### 3.5. The Relative Numbers of Lymphocytes and Monocyte in Peripheral Blood

Additionally, immune phenotypic analysis of monocyte and lymphocyte subpopulations was performed to estimate the impact of aging and/or AlCl_3_ treatment on the number of various immune cells in peripheral blood. Flow cytometry data did not demonstrate any statistically significant differences in the percentage of the general lymphocyte population (CD3^+^), including CD3^+^CD4^+^ T-helpers and CD3^+^CD8^+^ T-cytotoxic cells, among all observed groups (Table 1).

Meanwhile, the relative number of CD45R^+^ B-cells decreased 1.8 times in the Old-C group compared with the Adult-C one. In Adult-AlCl_3_ rats, it remained unchanged relative to Adult-C ones. At the same time, in the Old-AlCl_3_ group, it demonstrated a decrease of 2.6 times in comparison with the Adult-AlCl_3_ group, remaining at the same number as in the Old-C group.

**Figure 10:**
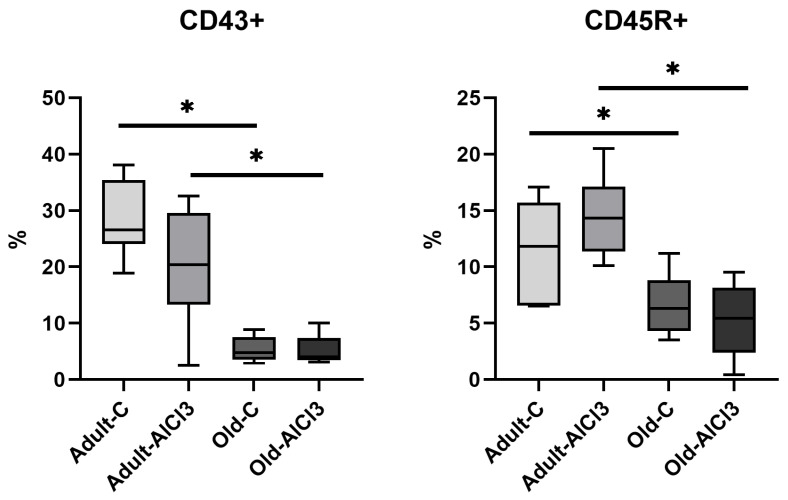
The percentage of CD45R^+^ B-cells and CD43^+^ monocyte in peripheral blood of adult and old Wistar rats of control and experimental groups. Adult-C—adult rats control group (n = 8); Adult-AlCl_3_—adult rats that consumed AlCl_3_ (n = 8); Old-C—old rats control group (n = 8); Old-AlCl_3_—old rats that consumed AlCl_3_ (n = 8). The data displayed as: line—median, box—25–75 quartiles, whiskers—nonoutlier range; *—*p* < 0.05. The Kruskal–Wallis test was used for multiple comparisons.

Likewise, the percentage of CD43^+^ monocyte was almost 5.5-fold higher in Adult-C rats in comparison with Old-C ones. No difference in this percentage, nor in the percentage of CD45R+ B-cells, was observed between Adult-C and Adult-AlCl_3_ groups. Old-AlCl_3_ animals showed a pronounced decline, of 5.2 times, compared with Adult-AlCl_3_ rats; no difference was observed in comparison with Old-C rats (Figure 10).

**Figure 11:**
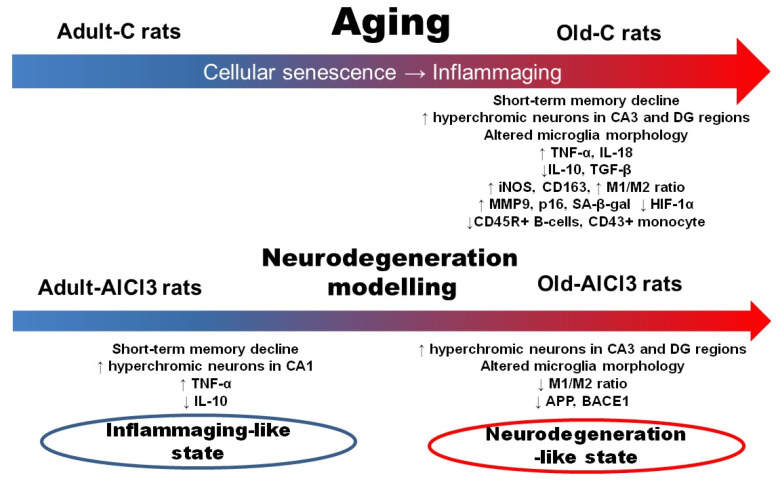
Morphofunctional changes in brain and peripheral blood in adult and old Wistar rats due to aging and neurodegeneration modelling. Adult-C—adult rats control group, Old-C—old rats control group, Adult-AlCl_3_—adult rats that consumed AlCl_3_, Old-AlCl_3_—old rats that consumed AlCl_3_, DG—dentate gyrus, TNF-α—tumor necrosis factor α, IL-18—interleukin 18, IL10—interleukin 10, TGF-β—transforming growth factor β, iNOS—inducible nitric oxide synthase, M1/M2 ratio—ratio of mRNA expression of markers for M1-type activated microglia to M2-type activated microglia, MMP9—matrix metallopeptidase 9, p16—cyclin-dependent kinase inhibitor 2A, Sa-β-gal—senescence-associated-β-galactosidase, HIF-1α—hypoxia-inducible factor 1α, APP—amyloid precursor protein, BACE1—β-Site APP-cleaving enzyme 1.

Hence, we observed a great deal of change among groups due to aging and neurodegeneration modelling (Figure 11). Comparing with the Adult-C group, Old-C rats showed (1) an increase in altered hyperchromic neurons’ percentage in CA3 and in the dentate gyrus of the hippocampus; (2) an alteration of microglia morphology and a shift in microglia from resting to activated states, both M1 and M2; (3) an upregulation of mRNA expression levels of pro-inflammatory cytokines (*Il-18* and *Tnf-a*), markers of microglial activation (*iNos* and *Cd163*) with M1-type predominance, and cellular senescence markers (*p16*, *Sa-β-gal*, and *Mmp9*); (4) a downregulation of anti-inflammatory cytokines’ mRNA expression levels (*Il-10* and *Tgf-β*) as well as *Hif-1a*; and (5) a decrease in the relative numbers of B-cells and monocyte in peripheral blood.

With exposure to AlCl_3_, Adult-AlCl_3_ rats demonstrated an increase in the percentage of hyperchromic neurons in the CA1 zone of the hippocampus alongside short-term memory decline, moderate upregulation of the *Tnf-α* expression level, and downregulation of the *Il-10* expression level.

At the same time, Old-AlCl_3_ animals displayed the greatest percentage of altered hyperchromic neurons, downregulation of *App* and *Bace1* mRNA expression, an alteration of microglia morphology to the dystrophic type, and a decline of M1/M2-activated microglia ratio.

## 4. Discussion

### 4.1. Morphofunctional Changes in Brain and Peripheral Blood of Wistar Rats Due to Aging

One of the main features of aging is cellular senescence manifesting in the increasing number of SASP-expressed cells. According to our data, old Wistar rats of the control group displayed this feature in the same way as it was observed in humans [14].

Our data obtained by MWM performance demonstrated a decline of short-term memory functions in old control animals relative to adult ones before the experiment began, although aged rats demonstrated escape latency similar to adult animals during the second session of trials. Apparently, aging affects the rat population highly heterogeneously, just as it does in humans, and we observed a high variability in the intertrial and interindividual results among all the animals, as colleagues did before [15]. This made statistical analysis and the interpretation of results quite a challenging task. However, it seems like multistage and highly interconnected memory-related processes tend to readjust with aging to sustain their efficacy [16], and some animals manage this better than other [17]. This could be correlated with the prevalence of lesions in the hippocampal region. 

We observed a notable increase in the percentage of altered hyperchromic neurons in the hippocampus of old control rats compared with adult ones. Recent data confirm an increase in the relative number of hyperchromic neurons in aged rats in comparison with adult animals without any external exposure due to aging itself [18]. In our work, we counted mostly morphologically altered neurons of lesser size and polygonal, shrunken and wrinkled shape. Apparently, the protein biosynthesis in such neurons was switched to providing for the needs of their own mostly, which is required for their survival in unfavorable conditions. However, due to the subsequent decrease in protein synthesis for export to synaptic terminals, these neurons’ activity and interconnections will inevitably fade [19]. The fate of hyperchromic neurons (recovery of functions or death) depends on the ongoing conditions as well as on the brain region. Ooigawa et al. reported lower survival rates of hyperchromic neurons in the hippocampus compared with those in the neocortex of rats that underwent traumatic brain injury [20]. Shrinkage of hyperchromic neurons probably demonstrates the degeneration and atrophy caused by the long-term persistence of unfavorable conditions, which could be regarded as a pathological state preceding cell death [21]. This should be interpreted as a severe pathological change, more permanent than not, with the persistence of particularly deleterious conditions. Hence, this shrinkage could be caused by the development of inflammaging, which is an unfavorable condition indeed. The upregulation of various pro-inflammatory cytokines and senescence biomarkers due to SASP expression contributes to the ROS production escalating, which is most harmful to mitochondria. With these “cell engines” damaged, it becomes even harder for neurons to maintain themselves properly in such an inauspicious environment, which leads to their maladaptation and eventual loss of functions.

Another factor which might contribute to neuronal dysfunction and, in particular, synaptic dysfunction, is microglia alteration. Our data of microglial morphology changing in old rats compared with adult ones are consistent with recent results obtained from humans. It was observed that identical changes of microglia appeared in healthy humans due to aging [12]. According to recent discoveries, microglia, which are resident immune cells in the CNS, might play a pivotal role in AD initiation and development [2]. Cellular senescence manifesting with SASP is responsible for the continuing increase in pro-inflammatory mediators’ production which, among other reasons, contributes to microglia activation and further establishes a vicious cycle of the disease [22]. It is also worth noting that microglial cells as well as all other types of cells, including immune ones, undergo the process of cellular senescence themselves [23], which additionally affects their features and disrupts their function. Shahidehpour et al. described similar age-related changes in microglia morphology, observed in healthy elderly people, as hypertrophic [12]. Apparently, long-term persistence of low-grade neuroinflammation made microglia primed for too long, and the microglia eventually become hypertrophic as well as senescent. As we showed, *Tnf-α* and *Il-18* mRNA expression levels rose dramatically in old rats relative to adult animals, which is consistent with previous researches [24]. These pro-inflammatory cytokines are produced mostly by microglia, although astrocytes and neurons are capable of secreting IL-18 as well [25], and perform essential pleiotropic effects under physiological conditions [25,26]. Although aging is also a type of physiological state, the more the lifespan is prolonged, the higher the number of SASP-expressing cells becomes. The imbalance that emerges between pro-inflammatory and anti-inflammatory mediators leads to the manifestation of age-related diseases [27]. This impact of inflammaging could also make microglia cells less susceptible to external signals, including anti-inflammatory mediators, and abate their immune surveillance and clearance functions.

Whereas the expression levels of pro-inflammatory mediators rose significantly in aged rats relative to adult animals, the levels of *Il-10* and *Tgf-β* demonstrated exactly the opposite. Like cytokines such as TNF-α and IL-18, they are involved in various integrative processes as well. So, IL-10 is able to inhibit the production of pro-inflammatory cytokines, e.g., TNF-α, ROS generation, microglia activation, and antigen presentation, by reducing the expression of the major histocompatibility complex class II (MHC II). It is also capable of enhancing B-cells’ proliferation and antibody production [28] and regulating neurogenesis [29] and production of anti-inflammatory mediators, mostly by glial cells, including TGF-β [30]. As for TGF-β itself, its pleiotropic impact includes the regulation of the differentiation of the resident cells of the CNS, in both the developing and adult brain [31], as well as synaptogenesis and synaptic transmission [32]. Apparently, it also has a neuroprotective effect, particularly against ischemia/hypoxia, and could be produced by both glial cells and neurons under such inauspicious conditions [33]. As an anti-inflammatory cytokine in the first place, TGF-β protects against collateral damages caused by the immune system. Furthermore, TGF-β is a prominent immune suppressor that inhibits the proliferation, differentiation, activation, and effector functions of immune cells, including microglia. Since microglia are a major source of TGF-β1 in the CNS, they might execute an auto-inhibitory control in this way [34]. All mentioned benevolent features of these cytokines occur under physiological conditions, whereas their permanent high level of expression due to cellular senescence and inflammaging [27,35] development becomes another deleterious factor [36]. A possible reason for why we registered no expression of *Il-10* and a dramatically decreased level of expression of *Tgf-β* is, on the one hand, the critical lack of cells’ energy supplies and switching metabolic processes in “power saving mode”, which could be inferred based on altered morphology of neurons.

On the other hand, when discussing microglia, the absent or lower levels of *Il-10* and *Tgf-β* expression could be a consequence of the activated state of the microglia, mostly M1. It is generally accepted that the CNS resident immune cells have “resting” and activated states. Microglia activation, as well as macrophage activation, leads cells to a pro-inflammatory M1 or anti-inflammatory M2 polarization state. There is also a continuum of different intermediate phenotypes between M1 and M2, and microglia can shift from one state to another depending on microenvironment condition changes [37]. So, it was not a surprise to observe the presence of a small percentage of both M1 and M2 microglia cells in healthy adult rodents. At the same time, there was a significant rise of *iNos* and *Cd163* expression levels, as well as an increase in the M1/M2 ratio and in the expression of pro-inflammatory cytokines, in old rats relative to adult ones. These data confirm the presence of a higher number of activated immune cells with M1 predominance caused by aging itself due to the development of SASP expression. Being both activated and senescent, these microglial cells not only fail in their clearance and immune surveillance functions, but also participate in the consistent aggravation of already existing SASP-established neuroinflammation.

According to our qPCR-RT data, aged but conditionally healthy rats have the same level of *App* and *Bace1* expression as healthy adult ones. APP is an integral membrane receptor widely distributed in most tissues. In the CNS, it has an impact on synaptogenesis and neuroplasticity processes. BACE1 is one of the secretases involved in its metabolism via the amyloidogenic pathway, but, according to recent studies, it also has a pivotal role in synaptic transmission, plasticity, and long-term potentiation vital for memory-related processes [38]. For decades, Aβ was regarded as the main culprit of AD, the most prevalent among neurodegenerative diseases. Apparently, however, the etiology and pathogenesis of AD are rather complicated, with various integral processes involved such as oxidative stress, microglial activation, inflammaging, etc. [22]. Aβ plagues are, indeed, the hallmark of the AD or Alzheimer’s continuum, but according to acting clinical guidelines, it is strictly a morphological diagnosis; AD as an organic disorder could exist in elderly people without any clinical manifestations [39]. There is a critical lack of data obtained from aged, non-transgenic rodents concerning changes in *App* and *Bace1* expression and subsequent protein biosynthesis. Therefore, we may only speculate that aging alone is probably not enough to disturb this precise part of neuronal metabolism and lead to the initiation of degeneration. Unlike laboratory rats, most people of advanced age have various diseases, including age-related diseases such as atherosclerosis, arterial hypertension, metabolic syndrome/obesity, T2DM, etc. All these pathological conditions are capable of enhancing the background of inflammaging. This confirms the multiplicity of mechanisms that initiate the processes of neurodegeneration [27]. In addition, it also provides evidence that most popular transgenic models of AD, the main feature of which is excessive APP production, do not provide a description of the disease development as it occurs in humans. The abnormally intensive APP synthesis in these mice causes the deposition of Aβ plagues in their brains and eventual cognitive impairments. However, these are both nonspecific clinical syndromes and an end point in the complex chain of previous physiological and pathological events. In this regard, Aβ or BACE1 inhibitor-based treatment could be considered only as a symptomatic therapy; mechanisms of initiation, lasting, developing, and triggering of the clinical manifestation of neurodegeneration cannot be extrapolated from transgenic mice to humans.

Aside from the aforementioned markers, we also observed a notable increase in *p16*, *Sa-β-gal*, and *Mmp9* mRNA expression levels and a downregulation of *Hif-1α*. As mentioned before, aging itself is a kind of physiological condition. It is inevitable that aging will appear as cellular senescence. In the first place, it is a means to prevent the development of tumors. With advanced aged, pro-oncogenes’ upregulation, anti-oncogenes’ downregulation, and irreparable accumulated DNA damage not only induce apoptosis, but also enhance the pace of cellular senescence to achieve this purpose. Although SASP manifestation is highly heterogeneous depending on tissue types, external stresses, and internal pathological processes [40], p16 and Sa-β-gal are regarded as its most universal markers [41]. p16 acts as a main inhibitor of several cyclin-dependent kinases (CDK) and arrests the cell cycle by blocking downstream processes and, therefore, the G1-S phase transition [35]. Occurrences such as the accumulation of DNA damage or the escalation of ROS production could promote its activation [42]. At the same time, Sa-β-gal is lysosomal β-galactosidase only in senescent cells. It is a less sensitive marker than p16 and a consequence of upstream processes leading to senescence rather than its substantive influencer [41,43]; however, it remains reliable evidence for the presence of SASP-expressed cells. Upregulation of these markers in aged animals was an expected find. MMP9 is a type IV collagenase involved in degradation of most structural elements of the extracellular matrix, which leads to both physiological and pathophysiological tissue remodeling. It is also one of the most common SASP components. Its expression could be upregulated by ROS both directly and indirectly through NF-κB pathway activation, which could happen due to an increase in *Tnf-a* as well [44]. In the CNS, microglia and macrophages migrated from peripheral blood are the main source of its production [45], and their activated state due to inflammaging is a reasonable explanation for *Mmp9* upregulation.

HIF-1α is a heterodimeric transcription factor mediating the adaptive response of mammalian cells to hypoxia. The higher its level, the more vulnerable cells will be to hypoxic conditions. Although HIF-1α is not considered as a marker of cellular senescence, apparently, its expression and accumulation in tissues is related with the stage of ontogenesis. Ndubuizu et al. reported a decline in HIF-1α protein, although not in mRNA expression, in the brain of aged Fischer 344 rats [46]. Colleagues from our department showed that *Hif-1α* was significantly lower in the liver of newborn and prepubertal male Wistar rats compared with adult ones, which confirms its fluctuation during the lifespan [47]. According to our recent yet unpublished data, its level of expression decreased notably in old male Wistar rats relative to adult ones. *Hif-1α* downregulation registered in old rats is probably another feature of adaptation adjustment due to aging, although further investigations in this regard are necessary.

Additionally, we observed a reduction of CD43^+^ monocyte and CD45R^+^ B-cells’ relative numbers in the peripheral blood of Old-C rats in comparison with Adult-C ones. A recent study investigating the impact of aging on lymphopoiesis in both humans and mice revealed a correlation between the rising production of TNF-α by peripheral B-cells and the inhibition of B-cell lymphopoiesis in the bone marrow [48]. Meanwhile, Snodgrass et al. detected a decrease in the circulating monocyte pool due to aging in humans [49]. This could happen due to the abating of monocytopoiesis in bone marrow as well as B-cells’ or cells’ destruction caused by inflammaging’s pro-inflammatory background; but it also could be explained by an intensification of monocyte migration to different tissues, including brain parenchyma, since inflammaging is a system condition involving the whole organism. It also could participate in an increase in *Tnf-α*, *Il-18*, and *Mmp9* expression levels in samples of brain tissue, since these cytokines themselves are involved in BBB integrity violation and the stimulation of monocyte chemotaxis, which form another loop in the vicious cycle of pathological processes.

### 4.2. Morphofunctional Changes in Brain and Peripheral Blood of Adult Wistar Rats with AlCl_3_-Induced Neurodegeneration

There are various transgenic and non-transgenic models of neurodegeneration, including Al^3+^ ions’ use. These ions are capable of increasing ROS production, which is involved in mitochondrial and DNA damage, promotion of pro-inflammatory mediators’ expression and secretion, and the development of a hypoxic condition. AlCl_3_-based models of AD, including those using a 100 mg/kg oral dosage, are described widely in the literature [50], and all these researches were conducted on adult rats and mice. Our task was to observe and evaluate distinctions in the reactions of adult and old rodents to the same external exposure.

We observed a significant increase in the escape latency during MWM acquisition trials in adult rats that had chronically consumed AlCl_3_ relative to control littermates, as previously reported [51], as well as the most notable damage in the CA1 zone of the hippocampus compared with CA3 and the dentate gyrus of adult rats of the control and experimental groups. This probably could be explained by the difference of susceptibility of hippocampal regions to hypoxia, which enhances inflammation and ROS activity increase mediated by Al^3+^. Apparently, CA1 neurons are more vulnerable to hypoxia and oxygen-glucose deprivation (ODG) than CA3 and DG [52], probably due to their higher activity [53]. CA1 is responsible for mediating the association with the temporal cortex and is capable of maintaining short-term memories, whilst CA3 is mostly involved in processes establishing rapid spatial and contextual memory [52]. Such function distinction makes CA1 neurons both more essential and energy dependent, which means they are more vulnerable to hypoxia. Notably, a similar change of neural density was observed in AD patients compared with healthy age-matched elderly people—it was the most severe in CA1 among the CA1-CA4 regions [54].

Relative to control littermates, adult rats that consumed AlCl_3_ demonstrated a statistically significant upregulation of *Tnf-α* expression, although not *Il-18* expression, as was reported earlier [55]. Since Al^3+^ is a strong promoter of ROS production enhancement, it inevitably leads to glial cell response and activation, with a subsequent increase in these mediators’ secretion. However, an increase in the absolute number of microglia or in the activated microglia percentage was not observed, which might explain such mild upregulation of pro-inflammatory cytokines. The statistically significant but moderate downregulation of *Il-10*, but not of *Tgf-β*, could also be a downstream event driven by just a faint shift of activated microglia continuum to the M1 state, as revealed by the M1/M2 ratio in the adult rats experimental group. Interestingly, both TNF-α and IL-18 might upregulate *App* and *Bace1* expression, including via the activation of the Nf-κB pathway [56], but the *Tnf-α* increase registered in rats of the experimental group was probably not adequately pronounced to impact these proteins’ metabolism.

Despite the ability of Al^3+^ to promote enhanced ROS production, which leads to oxidative stress and DNA damage, chronic exposure to Al^3+^ did not cause a notable increase in any cellular senescence markers. This possibly means that adult rats’ cells possess enough energy resources for adaptation to inauspicious environmental conditions and proper performance of their functions and self-maintenance, including DNA reparation.

With exposure to AlCl_3_, Adult-AlCl_3_ rats demonstrated a tendency to demonstrate a decrease in CD43^+^ monocyte percentage and an increase in CD45R^+^ B-cell percentage, although it was statistically insignificant. Regarding the aforementioned *Tnf-α* expression level increase, it could probably display a tendency for monocyte migration to the CNS. Considering that the data imply that an increase in TNF-α impacts negatively on B-lymphocytes proliferation [48,57], the increase seems more like an adaptive recruitment of B-cells from the spleen rather than an intensification of lymphopoiesis in the bone marrow.

### 4.3. Morphofunctional Changes in Brain and Peripheral Blood of Old Wistar Rats with AlCl_3_-Induced Neurodegeneration

All pathological processes described for old control rats are also valid for old animals that consumed AlCl_3_. These processes demonstrated the highest percentage of altered hyperchromic neurons, which was statistically significant in CA3 and DG relative to the adult rats’ experimental group. The tendency for it to increase appeared to apply to the old rats control group as well, although it was statistically insignificant. Since the pro-inflammatory background of inflammaging persisted in these animals before the beginning of the experiment, additional damaging impact of AlCl_3_ likely aggravated mitochondrial dysfunction, which led to severe energy shortage and neuronal loss.

Unlike in both Adult-AlCl_3_ and Old-C groups, microglia cells displayed signs of dystrophy in AlCl_3_–treated old rats instead of activation or hypertrophy features. It is highly likely that this is evidence of their maladaptation due to the harmful impact of AlCl_3_ and due to a critical shortage of self-maintenance resources, in the same way as it occurs in neurons. Such cells are unable to produce adequate amounts of proteins essential for proper functioning, as proved by qPCR-RT data, including the downregulation of *Cd163* and the decline in the M1/M2 ratio.

The expression level of *Il-18* was significantly higher in Old-AlCl_3_ rats in comparison with Adult-AlCl_3_ ones and tended to decline when compared with old control animals, whereas *Tnf-α* expression remained unchanged relative to both these groups. This could probably be related to neuronal and glial functions and activity decline due to the deleterious impact of AlCl_3_, on the one hand, and, on the other, due to peripheral monocyte migration to the CNS. Highly pronounced downregulation of both *Il-10* and *Tgf-β* might be caused by the same events as well.

Our data confirm that *App* and *Bace1* expression levels and, therefore, these proteins’ subsequent biosynthesis, decrease greatly as neurodegeneration progresses. This probably demonstrates the advanced stage of this detrimental process, when neurons are no longer capable of producing APP and BACE1 to form new synapses or maintain deteriorating ones.

Expression levels of cellular senescence markers likely remained unchanged in AlCl_3_-treated old rats because, due to aging itself, they reached the threshold of their upregulation.

Old-AlCl_3_ animals showed a significant decrease in both CD43^+^ monocyte’ and CD45R^+^ B-cells’ relative numbers in comparison with those in Adult-AlCl_3_ rats. At the same time, these levels remained the same as those in the Old-C group. Apparently, AlCl_3_ did not affect these peripheral immune cells’ destruction, proliferation, or migration, and their lesser percentage occurred due to aging before the beginning of the experiment. Since the pro-inflammatory background already persisted in old animals, and AlCl_3_ consumption did not cause its further development but a decrease in cytokine expression due to cell dystrophy and death, there were no microenvironmental conditions to change these immune cells’ percentage anyhow.

## 5. Conclusions

There are many clear and essential differences between adult and old rats’ physiological states. Unlike adult animals, old animals have the background of inflammaging as an aggravating factor alongside energy resource shortage caused by aging itself. Considering this, aged rats respond differently to aluminum exposure compared to young animals. Instead of adaptation to inauspicious conditions, they display a severe decline of most cells’ function and irreversible neuronal loss. Regarding all reported data, neurodegeneration modelling and investigation of the factors capable of accelerating or preventing this degeneration should be performed in experimental work on aged animals.

## Figures and Tables

**Table 1:** Relative number (%) of lymphocyte subpopulations in peripheral blood of adult and old Wistar rats of control and experimental groups (Me (25;75%)).

Peripheral Blood T-Cells, %	Adult-C	Adult-AlCl_3_	Old-C	Old-AlCl_3_	*p*
CD3^+^	25.8 (19.4–36)	29.25 (27.8–36.4)	23.35 (18.5–37.1)	20.9 (11.3–24.4)	>0.05
CD3^+^CD4^+^	11.3 (9.3–13.5)	13 (12.4–17.9)	12.75 (8.1–14.7)	11.3 (8.6–12.7)	>0.05
CD3^+^CD8^+^	12.3 (5.7–18.7)	12.1 (8–22.1)	12.65 (5.6–27.1)	6.2 (5.5–11.3)	>0.05

## Data Availability

Not applicable.

## Abbreviations

Adult-AlCl_3_	adult rats that consumed AlCl_3_
Adult-C	adult rats control group
AlCl_3_	aluminum chloride
APP	amyloid precursor protein
BACE1	β-Site APP-cleaving enzyme 1
BBB	blood–brain barrier
DG	dentate gyrus
CNS	central nervous system
GAPDH	glyceraldehyde 3-phosphate dehydrogenase
HIF-1α	hypoxia-inducible factor 1α
HRP	horseradish peroxidase
Iba1	ionized calcium binding adaptor molecule 1
ICH-P	Immunohistochemistry-paraffin protocol
IL-10	interleukin 10
IL-18	interleukin 18
iNOS	inducible nitric oxide synthase
MMP9	matrix metallopeptidase 9
MWM	Morris water maze
NF-κB	nuclear factor kappa-light-chain-enhancer of activated B cells
ODG	oxygen-glucose deprivation
Old-AlCl_3_	old rats that consumed AlCl_3_
Old-C	old rats control group
p16	cyclin-dependent kinase inhibitor 2A
qPCR-RT	quantitative polymerase chain reaction, real-time regime
ROS	reactive oxygen species
Sa-β-gal	senescence-associated β-galactosidase
SASP	senescence-associated secretory phenotype
TGF-β	transforming growth factor β
TNF-α	tumor necrosis factor α

## Appendix A

**Table A1:** qPCR-RT primers sequences used (all selected precisely for each rat species).

Primer	Forward Sequence	Reverse Sequence
GAPDH	GCGAGATCCCGCTAACATCA	CCCTTCCACGATGCCAAAGT
IL-18	GACAAAAGAAACCCGCCTG	ACATCCTTCCATCCTTCACAG
TNF-a	CCACCACGCTCTTCTGTCTA	GCTACGGGCTTGTCACTCG
IL-10	GCCCAGAAATCAAGGAGCAT	TGAGTGTCACGTAGGCTTCTA
TGF-β	CCGCAACAACGCAATCTATG	AGCCCTGTATTCCGTCTCCTT
iNOS	CGCTGGTTTGAAACTTCTCAG	GGCAAGCCATGTCTGTGAC
CD163	TCTTGTGGACTCTGAAGCGA	TCTTAAATGCCAACCCGAGG
APP	TGGATGATCTCCAACCGTG	CGTCGACAGGCTCAACTTC
BACE1	GGGCAGTAGTAATTTTGCAGT	TTCGGAGGTCTCGGTATGT
p16	GTACCCCGATACAGGTGATG	GGTGCAGTACTACCAGAGTG
Sa-β-Gal	CTTCCGGATACCCCGATTCT	AGGGCACGTACGTCTGGAT
MMP9	ATGGTTTCTGCCCCAGTGAG	CACCAGCGATAACCATCCGA
HIF-1a	TCACAGTCGGACAACCTCAC	TGCTGCAGTAACGTTCCAATTC
